# Low prevalence of neuropathic-like pain symptoms in long-term controlled acromegaly

**DOI:** 10.1007/s11102-021-01190-z

**Published:** 2021-10-23

**Authors:** Victoria R. van Trigt, Iris C. M. Pelsma, Herman M. Kroon, Alberto M. Pereira, Coen van der Meulen, Margreet Kloppenburg, Nienke R. Biermasz, Kim M. J. A. Claessen

**Affiliations:** 1 Department of Medicine, Division of Endocrinology, and Center for Endocrine Tumors Leiden, Leiden University Medical Center, Albinusdreef 2, 2333 ZA Leiden, The Netherlands; 2 Department of Radiology, Leiden University Medical Center, Leiden, The Netherlands; 3 Department of Rheumatology, Leiden University Medical Center, Leiden, The Netherlands; 4 Department of Epidemiology, Leiden University Medical Center, Leiden, The Netherlands

**Keywords:** Acromegaly, GH/IGF-1, Pain, Neuropathic pain, HR-QoL

## Abstract

**Purpose:**

Pain is a common symptom of acromegaly, impairing health-related quality of life (HR-QoL) significantly despite long-term disease remission. Neuropathic-like pain (NP-like) symptoms are invalidating, with great impact on HR-QoL. Studies characterizing or investigating the etiology of pain in acromegaly are scarce. Therefore, we aimed to assess NP-like symptoms in a cohort of controlled acromegaly patients.

**Methods:**

Forty-four long-term controlled acromegaly patients (aged 62.6 ± 12.6 years; 56.8% female) were included in this cross-sectional study. NP-like symptoms were assessed using the validated painDETECT questionnaire. Patients were divided in three probability-based NP-like symptoms categories based on the total score (range 0–35): unlikely (≤ 12), indeterminate (13–18) and likely (≥ 19). HR-QoL (physical component score (PCS), and mental component score (MCS)), and self-reported pain were assessed using Short Form-36 (SF-36). Potential risk factors were determined using linear regression analyses.

**Results:**

Self-reported pain was reported by 35 patients (79.5%). Likely NP-like symptoms were present in 4/44 patients (9.1%), and indeterminate NP-like symptoms in 6/44 patients (13.6%). All patients with likely NP-like symptoms were female. Higher painDETECT scores were negatively associated with HR-QoL (PCS: r = − 0.46, P = 0.003; MCS: r = − 0.37, P = 0.018), and SF-36 pain scores (r = − 0.63, P < 0.0001). Female sex was a risk factor for NP-like symptoms.

**Conclusions:**

Pain was prevalent in controlled acromegaly patients, whereas NP-like symptoms were relatively infrequent, and only observed in females. NP-like symptoms were associated with lower HR-QoL in acromegaly. Since specific analgesic therapy is available, awareness for characterization, increased understanding, and clinical trials regarding neuropathic pain identification and treatment in acromegaly patients are warranted.

**Supplementary Information:**

The online version contains supplementary material available at 10.1007/s11102-021-01190-z.

## Introduction

Acromegaly is associated with an abundance of partially irreversible symptoms and comorbidities, significantly contributing to decreased health-related quality of life (HR-QoL) [1], which improves after acromegaly treatment albeit without complete normalization [2]. Pain is a prominent and invalidating feature—self-reported by 73% of patients 10 years after initial therapy [3–5]—reported more frequently compared to age-, and gender-matched controls [6]. Several acromegaly-associated musculoskeletal comorbidities may cause pain, e.g. carpal tunnel syndrome (prevalence: 18–84%) [7, 8], arthropathy characterized by cartilage hypertrophy and severe osteophytosis (prevalence: 72–94%) [1, 9–11], and vertebral fractures (prevalence: 60%) [12–14]. Additionally, headaches are frequently reported by patients due to either mechanical adenoma pressure, or growth hormone (GH) excess (prevalence: 37–87%) [8, 15].

Considering the diverse etiology of pain in acromegaly patients, the origin, and type of pain are difficult to determine. Generally, pain can be divided into two types: nociceptive pain caused by activation of nociceptors, and neuropathic pain resulting from lesions/diseases of the peripheral somatosensory nervous system [16]. Neuropathic pain is considered more invalidating than nociceptive pain [17, 18], being associated with depression and lower HR-QoL [18]. Moreover, several acromegaly-associated comorbidities might increase the risk for neuropathic pain, e.g. diabetic neuropathy, and mechanical complaints (*incl.* compression neuropathies) [4, 7, 8, 19–21]. Since specific analgesic therapy, e.g. tricyclic antidepressants, is indicated for neuropathic pain, adequate neuropathic pain identification is warranted to optimally address pain in patients with acromegaly [22, 23].

To our knowledge, only one previous study investigated neuropathic pain in pituitary adenoma patients, including acromegaly, of whom 20% reported neuropathic-like pain symptoms [3]. In the present study, we investigated the prevalence of neuropathic-like pain symptoms, potential associated clinical determinants, and the relationship between neuropathic-like pain symptoms and HR-QoL in a cohort of controlled acromegaly patients.

## Materials and methods

### Study design and patient selection

#### Study design

All patients gave informed consent for this cross-sectional observational study, which was approved by the Medical Ethics Committee. A standardized questionnaire on medical history, and current health condition was completed, including the past and current use of medication. Serum samples were obtained to assess pituitary function. Validated questionnaires, including a specific neuropathic pain symptom questionnaire, were filled out (vide infra).

#### Patients

Forty-four patients with well-controlled acromegaly with complete neuropathic pain questionnaires were included in the present study. Twenty-five patients in remission that participated in our long-term follow-up study on musculoskeletal acromegalic complications (using data from the last study visit—details published prior [13, 24]) were combined with nineteen additional patients in remission that were consecutively included at the time of the last study visit from new referrals to our expertise center.

### Study parameters

#### Acromegaly disease parameters and pituitary function

Clinical assessment of patients with acromegaly has been described previously [1, 9–11, 25–31]. Briefly, disease remission was defined as normal glucose-suppressed serum GH and IGF-1 levels (corrected for age and sex) [1, 9–11, 16, 25–31]. Hypopituitarism was treated according to available guidelines [13, 24, 32].

#### Assays

Serum GH levels were measured using a nationally harmonized assay (IDS-iSYS immunoanalyzer; harmonization factor: 1.02) [33]. For IGF-1, serum levels (IDS-iSYS immunoanalyzer), and SDS scores were used (based on age- and sex-dependent λ-μ-σ smoothed reference curves) [34, 35].

#### Questionnaires

Patients filled out all questionnaires based on their health status at the moment of the study visit. Notably, because of the observational nature of the study, the current prescribed treatments, and medications were continued.

##### Neuropathic pain

The 9-item *painDETECT Questionnaire* (painDETECT) was validated to identify neuropathic-like pain symptoms in various conditions including lower back pain, rheumatoid arthritis, and primary OA [36–41]. The painDETECT questionnaire (in English) is shown in Supplementary File 1. The painDETECT consists of seven questions on different aspects of neuropathic pain (7-item version), and two additional questions on pain localization and course over time (9-item version) (vide infra). Examples of questions are “Do you have a tingling or prickling sensation in the area of your pain (like crawling ants or electrical tingling)?”, and “Does slight pressure in this area, e.g. with a finger, trigger pain?”. Total painDETECT scores based on the 7-item version of the painDETECT ranged from 0 to 35 (7 questions using a 6-point Likert scale from 0 to 5) [42]. Based on the total painDETECT scores, patients were divided into three groups, reflecting the probability of neuropathic-like pain symptoms: unlikely-NP, total score ≤ 12; indeterminate-NP, total score 13–18; likely-NP, total score ≥ 19 [43, 44]. Outcomes of the remaining two questions of the 9-item painDETECT on course of pain over time, and pain radiation were available for a subset of 35, and 37 patients, respectively.

**Fig. 1:**
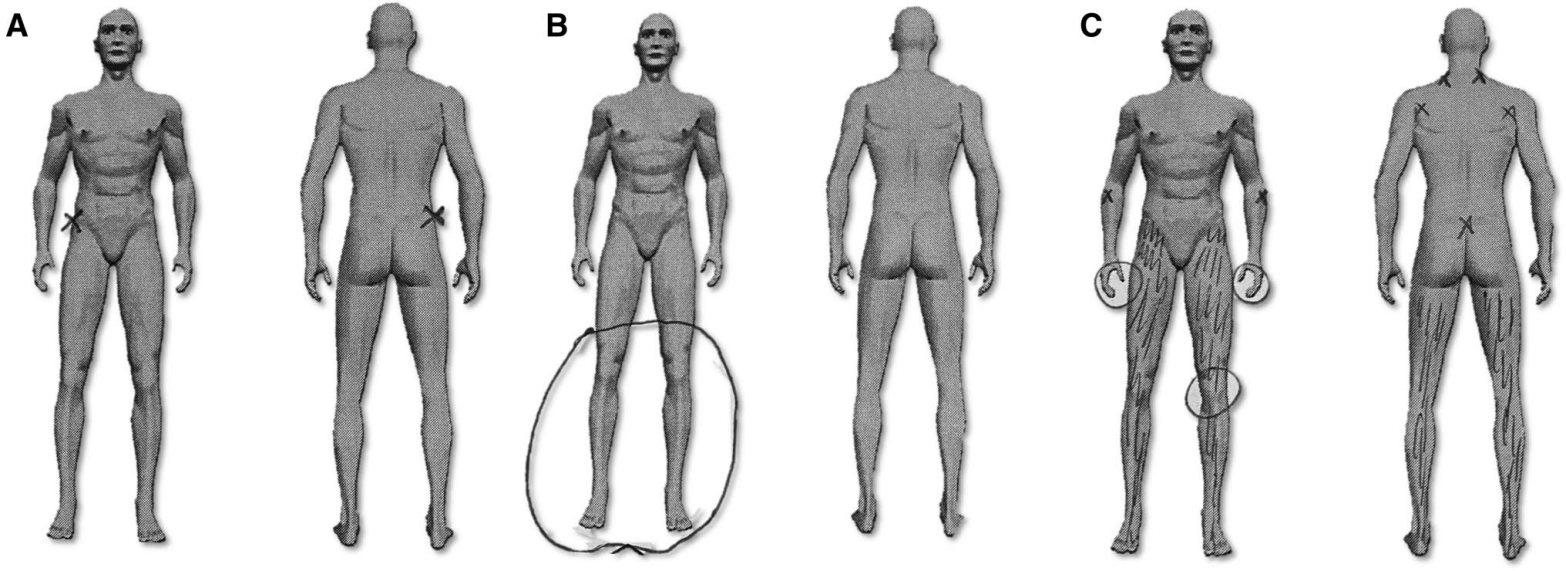
Examples of different categories of pain localization based on drawings in controlled acromegaly patients. Examples of the pain localization as assessed by drawings of individual controlled acromegaly patients on the human figure on the painDETECT questionnaire. Three of four categories were observed in our patients: **A** pain in one specific location, **B** pain in two separate limbs, and **C** pain diffusely spread throughout the body

Additionally, information on pain localization was extracted from the drawings of individual patients on the human figure within the painDETECT questionnaire. Data were available for 31 patients. Pain localization was divided into 4 categories: (1) pain in one specific location, (2) pain in one limb, (3) pain in two separate limbs, and (4) pain diffusely spread throughout the body (Fig. 1).

##### Joint symptoms

All patients completed the following questionnaires: *Australian/Canadian Osteoarthritis Hand Index (*AUSCAN) to measure hand disability [45]; *Disabilities of the Arm, Shoulder and Hand* (DASH) to assess disability of the upper limb [46–48]; *Western Ontario and McMaster Universities Arthritis Index *(WOMAC) to evaluate lower limb joint symptoms [45, 49]. In detail, the AUSCAN questionnaire [45]—used for evaluation of hand symptoms in the last 48 h—rates all items on a 5-point Likert scale ranging from 0 (none) to 4 (extreme), and divided items in three categories: pain (scores ranging from 0 to 20), stiffness (range 0–4), and joint function (range 0–36), with total AUSCAN scores ranging from 0 to 60. The DASH questionnaire [46–48, 50]—used to assess symptoms and function of the upper limbs in the last 7 days—rates 30 questions on a 5-point Likert scale. Following transformation, total scores ranged from 0 to 100. The WOMAC questionnaire [51]—used to assess disability of the lower limb—rates all items on a 100 mm visual analog scale (VAS), with 0 representing the absence of complaints, and 100 the worst score possible. Total WOMAC scores ranged from 0 to 300, with subscores (pain, stiffness, and function) ranging from 0 to 100. For all questionnaires, higher total scores indicated greater disability.

##### Health-related QoL

Patients completed the HR-QoL questionnaire *Short Form-36* (SF-36) [52]. Physical health component score (PCS), mental health component scores (MCS), and pain component scores were calculated [53], with higher scores indicating better HR-QoL (total score 0–100) [54]. Based on the SF-36 question on presence of pain (ranging from absence to very severe), current presence of pain was defined as any answer but absence (dichotomous).

### Statistical analysis

SPSS for Windows version 25.0 (SPSS Inc., Chicago, IL, USA) was used for data analysis. Data are presented as N (%), mean ± SD, or median (IQR). Pearson’s correlation analyses and linear regression analysis using numerical 7-item painDETECT total scores were performed. P-values < 0.05 were considered significant.

## Results

### Patient characteristics

Forty-four patients with controlled acromegaly (mean age 62.6 ± 12.6 years, 56.8% female) were included, as summarized in Table 1. Patients were in remission for a median of 17.1 years (IQR 7.3–25.4), with 18 patients currently receiving pharmacological treatment. Ten patients received somatostatin (SMS) analogue monotherapy, three patients received Pegvisomant (PegV) monotherapy, three patients were treated with SMS analogue and PegV combination therapy, and two patients were treated with SMS analogue and dopamine agonist combination therapy. Six patients (13.6%) had diabetes mellitus. Ten patients (22.7%) currently used medication potentially affecting neuropathic pain (e.g., serotonin reuptake inhibitors, tricyclic antidepressants, pramipexol, sumatriptan or opioids).

**Table 1:** General characteristics of the patient population

Clinical characteristics		All patients N = 44
*Demographic features*		
Sex (female)		25 (56.8%)
Age (years)		62.6 ± 12.6
Body mass index (kg/m^2^)*		28.1 ± 5.4
*Treatment features*		
Duration of active disease (years)*		8.2 ± 6.7
Duration of remission (years)**		17.1 (IQR 7.3–25.4)
Hypopituitarism		17 (38.6%)
	GH deficiency	9 (20.5%)
	Hypogonadism	9 (20.5%)
	Hypocortisolism	9 (20.5%)
	Hypothyroidism	11 (25.0%)
	Diabetes insipidus	4 (9.1%)
Type of treatment		
	PharmaT	5 (11.4%)
	Surgery	18 (40.9%)
	Surgery + PharmaT	13 (29.5%)
	Surgery + RT	5 (11.4%)
	Surgery + RT + PharmaT	2 (4.5%)
	RT + PharmaT	1 (2.3%)
*Biochemical characteristics*		
GH (μg/L) GH (μg/L)		
	Pre-treatment***	30.8 (IQR 15.0–53.5)
	Current****	1.4 (IQR 0.6–5.1)
IGF-1 (nmol/L)		
	Pre-treatment****	68.7 ± 28.3
	SDS****	6.5 ± 2.9
	Current*	16.8 ± 4.9
	SDS*	0.6 ± 1.0

Data is reported as N (%), mean ± SD, or median (IQR). Currently, eighteen patients received pharmaT; SMS analogue monotherapy (N = 10), PegV monotherapy (N = 3), SMS analogue and PegV combination therapy (N = 3), and SMS analogue and dopamine agonist combination therapy (N = 2)

*GH* growth hormone, *IGF-1* insulin-like growth factor-1, *PegV* Pegvisomant, *PharmaT* pharmacological treatment, *RT* radiotherapy, *SMS* somatostatin

*Data available in 40 patients

**Data available in 41 patients

***Data available in 21 patients

****Data available in 31 patients

### Pain

Pain was reported by 35 patients (79.5%), and median SF-36 pain component scores were 68.4 (IQR 57.1–89.8). Pain medication was used by 12 patients (i.e. paracetamol N = 8; nonsteroidal anti-inflammatory drugs N = 4). Median painDETECT total scores were 5 (IQR 2–12), as shown in Table 2. Based on painDETECT total scores, four patients (9.1%) likely suffered from neuropathic-like pain symptoms, six patients (13.6%) indeterminately suffered from neuropathic-like pain symptoms, and 34 patients (77.3%) were unlikely to suffer from neuropathic pain. All four patients with likely-NP were female (P = 0.186).

**Table 2:** Characteristics of neuropathic-like pain symptoms

Neuropathic pain-like symptoms		All patients N = 44
Total painDETECT score		5 (IQR 2–12)
NP-like symptoms categories	Likely	4 (9.1%)
	Indeterminate	6 (13.6%)
	Unlikely	34 (77.3%)
*Course of pain, radiation and localization*		
Course of pain*	Continuous pain with slight fluctuations	14 (40.0%)
	Continuous pain with pain peaks	4 (11.4%)
	Pain-free periods with pain peaks	14 (40.0%)
	Continuous pain with extreme fluctuations	3 (8.6%)
Radiating pain**		14 (37.8%)
Pain localization***	One specific location	8 (25.8%)
	One limb	0 (0.0%)
	Two separate limbs	16 (51.6%)
	Diffusely spread throughout the body	7 (22.6%)

Outcomes of the validated painDETECT questionnaire on neuropathic-like pain symptoms are shown, of which the total scores range from 0 to 35. Neuropathic-like pain symptom categories were unlikely-NP, total score ≤ 12; indeterminate-NP, total score 13–18; likely-NP, total score ≥ 19 [43, 44]. Data is reported as N (%), or median (IQR)

*IQR* interquartile range, *NP* neuropathic pain

*Data available in 35 patients

**Data available in 37 patients

***Data available in 31 patients

Three of four likely-NP patients suffered from relevant comorbidities: demyelinating polyneuropathy (N = 1), diabetes mellitus (N = 1), and spondylolisthesis (N = 1). Of the six patients with indeterminate neuropathic-like pain symptoms, two patients suffered from a disc herniation, one patient had diabetes mellitus, and one patient had multiple sclerosis. Twelve unlikely-NP patients suffered from relevant comorbidities (viz*.* diabetes mellitus, polyneuropathy, carpal tunnel syndrome, brachial plexopathy, disc herniation, neural foraminal stenosis, spinal cord injury). When assessing whether the locations of pain from these comorbidities overlapped with the regions of pain indicated on the painDETECT questionnaire, overlap of the painful regions was observed in four patients (total N = 1, and partial N = 3), whereas no overlap was observed in three patients.

Out of the ten indeterminate, and likely NP patients, four patients used medication potentially affecting neuropathic pain-like symptoms: two likely-NP patients (SSRI prescribed for depression (N = 1), amitriptyline prescribed for neuropathic pain caused by demyelinating polyneuropathy), and two indeterminate-NP patients (SSRIs prescribed for depression (N = 2)).

### Relationship between painDETECT, self-reported joint symptoms and QoL

**Fig. 2:**
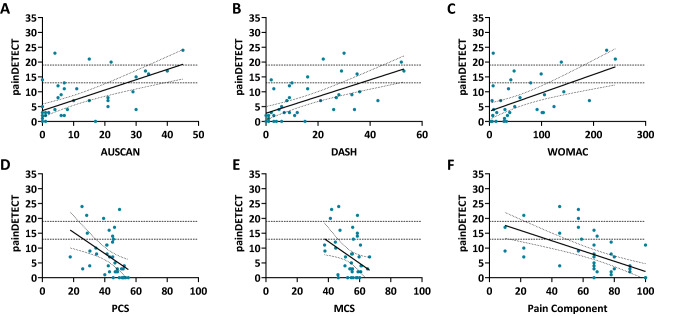
Correlations between painDETECT scores and OA-related disability and HR-QoL. Correlations between painDETECT scores and AUSCAN, DASH, WOMAC, MCS, PCS, and pain component scores are depicted. Horizontal dashed lines at painDETECT values 13 and 19 indicating indeterminate and likely, respectively, neuropathic-like pain symptoms cut-off points. **A** Correlation between painDETECT and AUSCAN scores (r = 0.62, P < 0.0001), **B** correlation between painDETECT and DASH scores (r = 0.63, P < 0.0001), **C** correlation between painDETECT and WOMAC scores (r = 0.56, P < 0.0001), **D** correlation between painDETECT and PCS (r = − 0.46, P = 0.003), **E** correlation between painDETECT and MCS (r = − 0.37, P = 0.018), **F** correlation between painDETECT and pain component score (r = − 0.63, P < 0.0001). *AUSCAN* Australian/Canadian Osteoarthritis Hand Index, *DASH* Disabilities of the Arm, Shoulder and Hand, *WOMAC* Western Ontario and McMaster Universities Arthritis Index, *MCS* mental health component scores, *PCS* physical health component score

Higher painDETECT scores were correlated with lower HR-QoL (PCS: r = − 0.46, P = 0.003; MCS: r = − 0.37, P = 0.018, respectively), especially with the pain component scores (r = − 0.63, P < 0.0001), as depicted in Fig. 2. In addition, higher painDETECT scores were associated with worse WOMAC (r = 0.56, P < 0.0001), AUSCAN (r = 0.62, P < 0.0001), and DASH scores (r = 0.63, P < 0.0001) (Fig. 2).

### Risk factors for neuropathic pain

The effects of age, sex and BMI, all being factors known to influence neuropathic pain in the general population [17], were assessed, and solely female sex was significantly associated with neuropathic-pain like symptoms (β = 5.24 ± 2.22, P = 0.024). Following adjustment for sex, painDETECT scores were not associated with pre-treatment IGF-1 levels (β = − 0.05 ± 0.05, P = 0.344), current GH levels (β = − 0.00 ± 0.01, P = 0.586), treatment modality (viz*.* surgery and radiotherapy *vs* pharmacological treatment) (β = − 0.40 ± 2.05, P = 0.848), active disease duration (β = 0.21 ± 0.16, P = 0.192), remission duration (β = 0.03 ± 0.10, P = 0.770), or hypopituitarism (β = 2.72 ± 2.20, P = 0.223).

### Pain radiation and localization

Radiation of pain could be assessed in 37 patients (Table 2). Radiating pain was present in 3 out of 4 patients with likely-NP, 2 out of 5 patients with indeterminate-NP, and 9 out of 28 patients with unlikely-NP patients, respectively. Course of pain could be assessed in 35 patients (Table 2). Three out of 4 patients with likely-NP patients experienced continuous pain with extreme fluctuations. Indeterminate-, and unlikely-NP patients reported generally pain-free periods with pain peaks, or continuous pain with slight fluctuations.

In addition, 31 patients provided drawings to assess pain localization. None of the patients reported pain in one limb (category 2), whereas examples of the other categories are shown in Fig. 1. Twenty-eight of 31 patients localizing pain on the painDETECT questionnaire also reported the presence of pain on the HR-QoL questionnaire. In these 28 patients, pain was localized in one specific site in 6 patients, in two separate limbs in 15 patients, and diffusely spread throughout the body in 7 patients. The remaining 3 patients that did not report pain on the HR-QoL questionnaire, indicated some form of pain in either one specific location (N = 2), or two separate limbs (N = 1).

## Discussion

The present explorative, questionnaire-based study reported that, despite a high prevalence of pain in controlled acromegaly patients, neuropathic-like pain symptoms are a relatively infrequent finding, and occurred in females only. Patients with higher painDETECT scores, reflecting a higher probability of neuropathic-like pain symptoms, reported lower HR-QoL, and more OA-related disability. All but two patients with likely or indeterminate neuropathic-like pain symptoms were diagnosed with relevant comorbidities. Apart from female sex, no other (acromegaly-specific) factors were associated with neuropathic-like pain symptoms in our cohort.

Neuropathic pain is caused by altered sensory signal transduction to the brain due to lesions or diseases of the somatosensory nervous system. Symptoms typically include burning, tingling sensations (dysesthesia), and pain upon non-painful stimuli (allodynia) [55]. In the general population, neuropathic-like pain symptoms are more frequent in women (8.0% in females *vs* 5.7% in males), and individuals > 50 years of age [17], which strengthens our findings that reported neuropathic-like pain symptoms were worse, and experienced specifically in female acromegalics. Notably, the increased reporting of pain in females is not specific for neuropathic pain, since females report more pain in general, including OA-related pain [56, 57].

In the present study, some form of pain was self-reported by over 75% of patients with controlled acromegaly, which is in line with previously reported prevalence [3]. However, although pain is a prevalent symptom in acromegaly, neuropathic-like pain symptoms were likely in only 10% of patients, and potentially observed in 25% of patients (i.e. likely-NP in 10%, and indeterminate-NP in 15% of patients). The prevalence of neuropathic-like pain symptoms in our population is slightly higher than the sole other study on neuropathic pain in pituitary adenoma patients, reporting that neuropathic pain was likely in approximately 5% of acromegaly patients using the same painDETECT questionnaire [3]. In addition to the aforementioned study, two case reports have reported on the invalidating potential of (severe) neuropathic pain in acromegaly [20, 58], highlighting the need for additional studies on this topic. The prevalence of neuropathic pain in our study among long-term controlled acromegaly patients was comparable with the general population (7 to 10%) [59], which was quite unexpected as acromegaly is associated with multiple typical neuropathic pain conditions, e.g. diabetic neuropathy, and compression neuropathies [4, 7, 8, 19–21]. Moreover, acromegaly patients have a high prevalence of arthropathy that partly resembles primary OA, and it is known from studies among patients with knee and hip OA that the prevalence of neuropathic-like pain symptoms is 23% [60]. The controlled status of included acromegaly patients might partially explain the low prevalence of neuropathic-like pain symptoms in our controlled patient population, since treatment was shown to significantly decrease neuropathic symptoms (e.g. paresthesia) in a meta-analysis of 24 studies [2].

Furthermore, we assessed the localization of pain, and presence of pain radiation. The localization of pain was limited to one specific location or joint in 25% of patients, with 75% patients localizing their pain in multiple limbs and joints, or present diffusely throughout the entire body. Furthermore, radiating pain—a phenomenon whereby pain of one specific origin is perceived in other locations—was observed in approximately 40% of patients. Since radiating pain is one of the characteristics of neuropathic pain, presence of pain radiation might underline the existence of, or risk to develop neuropathic pain in the future.

Higher upper and lower limb disability scores, and lower mental and physical HR-QoL were observed in patients with (potential) neuropathic-like pain symptoms. These observations are in line with general population studies showing that neuropathic pain is generally more invalidating than nociceptive pain [17, 18], and is thereby significantly associated with depression and poorer HR-QoL [3, 18]. The sole risk factor for neuropathic-like pain symptoms in our study with controlled acromegaly patients was female sex, whilst age, and BMI (factors known to influence neuropathic pain in the general population [17]) were not observed in the current patient population. Measures for acromegalic disease activity (e.g. GH and IGF-1 levels, and disease duration) did not influence the risk of neuropathic-like pain symptoms. Therefore, we assume that neuropathic pain and neuropathic-like pain symptoms in acromegaly are likely to be caused by acromegalic comorbidities (e.g. neuro-toxic metabolic dysregulation, or compression neuropathies), and not directly by GH and IGF-1 excess. Moreover, the presence of neuropathic-like pain symptoms could be explained by neurological comorbidities not associated with acromegaly, e.g. disc herniation, and multiple sclerosis [61].

**Fig. 3:**
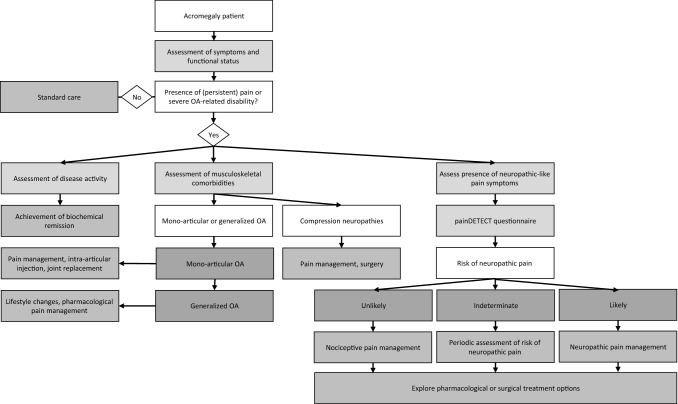
Flowchart of decision-making regarding pain diagnostics and management in acromegaly patients. Proposed algorithm for the diagnosis, and treatment for pain in acromegaly, including the assessment of the presence of neuropathic = like pain symptoms using the painDETECT questionnaire. *OA* osteoarthritis

Based on the findings of our study, and the scarcely available literature on neuropathic pain in the general population, we have constructed an algorithm for the diagnostics and management of pain in acromegaly, summarized in Fig. 3. In the case of a patient with pain, or OA-related disability, we advise to assess disease activity, and presence of associated comorbidities (e.g. OA, compression neuropathies, and vertebral fractures). Moreover, we advise to assess neuropathic-like pain symptoms using a self-administered questionnaire in patients to determine the likelihood of neuropathic and nociceptive pain, and to ensure adequate pain treatment, as neuropathic pain requires specific analgesic therapy with anticonvulsants and tricyclic antidepressants [22, 23].

Several limitations need to be addressed. Notably, outcomes of the SF-36 pain component and painDETECT questionnaire were partially non-overlapping, resulting in a potential higher prevalence of pain of over 85% in the present population. Moreover, as the study cohort was small, numerical questionnaire outcomes were used for analyses, whereas the painDETECT questionnaire was originally developed to have a categorical outcome. Additionally, presence of neuropathic-like pain symptoms was solely determined based on the painDETECT questionnaire, as clear, objective criteria for identifying neuropathic pain remain unavailable. Furthermore, the origin of neuropathic pain in acromegaly is difficult to determine, as multiple painful complications may coexist. Lastly, the present patient population included controlled acromegaly patients only, whereas patients with active disease might be more severely affected with regards to neuropathic (pain) symptoms.

In conclusion, although pain is a prevalent symptom in acromegaly patients, the infrequently occurring neuropathic-like pain symptoms—only found in females—suggests that pain in controlled acromegaly mostly is of nociceptive nature. The presence of neuropathic-like pain symptoms was associated with lower mental and physical HR-QoL and more OA-related disability. We advise to assess for neuropathic-like pain symptoms in patients with compromised HR-QoL, and severe, persisting pain despite regular pain medication.

## Supplementary Information

Below is the link to the electronic supplementary material.

Supplementary file1 (PDF 141 kb)

## Data Availability

Available upon request.
