# Supplementary material for: Low prevalence of neuropathic-like pain symptoms in long-term controlled acromegaly
Source: Pituitary. 2021 Oct 23;25(2):229–37. doi: 10.1007/s11102-021-01190-z (PMC8894222; doi:10.1007/s11102-021-01190-z)
Supplement: Supplementary file 1 — Supplementary file1 (PDF 141 kb) [file 11102_2021_1190_MOESM1_ESM.pdf]

PAIN QUESTIONNAIRE

painDETECT

Date: ____________ **Patient:** **Last name:** ______________ **First name:** ______________

How would you assess your pain **now**, at this moment?

0	1	2	3	4	5	6	7	8	9	10

none — max.

How strong was the **strongest** pain during the past 4 weeks?

0	1	2	3	4	5	6	7	8	9	10

none — max.

How strong was the pain during the past 4 weeks **on average**?

0	1	2	3	4	5	6	7	8	9	10

none — max.

Mark the picture that best describes the course of your pain:

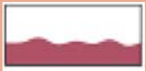

Persistent pain with slight fluctuations ☐

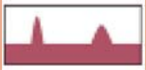

Persistent pain with pain attacks ☐

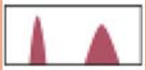

Pain attacks without pain between them ☐

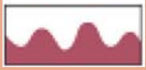

Pain attacks with pain between them ☐

Please mark your
main area of pain

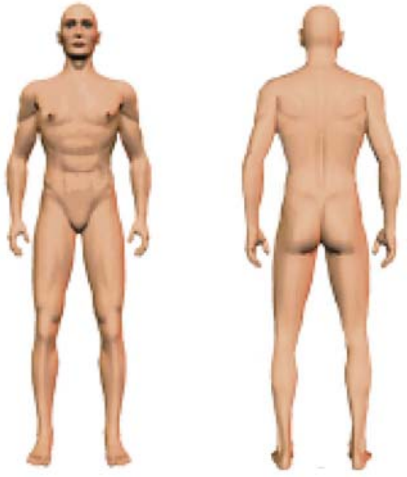

Does your pain radiate to other regions of your body? yes ☐ no ☐

If yes, please draw the direction in which the pain radiates.

Do you suffer from a burning sensation (e.g., stinging nettles) in the marked areas?

never ☐ hardly noticed ☐ slightly ☐ moderately ☐ strongly ☐ very strongly ☐

Do you have a tingling or prickling sensation in the area of your pain (like crawling ants or electrical tingling)?

never ☐ hardly noticed ☐ slightly ☐ moderately ☐ strongly ☐ very strongly ☐

Is light touching (clothing, a blanket) in this area painful?

never ☐ hardly noticed ☐ slightly ☐ moderately ☐ strongly ☐ very strongly ☐

Do you have sudden pain attacks in the area of your pain, like electric shocks?

never ☐ hardly noticed ☐ slightly ☐ moderately ☐ strongly ☐ very strongly ☐

Is cold or heat (bath water) in this area occasionally painful?

never ☐ hardly noticed ☐ slightly ☐ moderately ☐ strongly ☐ very strongly ☐

Do you suffer from a sensation of numbness in the areas that you marked?

never ☐ hardly noticed ☐ slightly ☐ moderately ☐ strongly ☐ very strongly ☐

Does slight pressure in this area, e.g., with a finger, trigger pain?

never ☐ hardly noticed ☐ slightly ☐ moderately ☐ strongly ☐ very strongly ☐

(To be filled out by the physician)

never	hardly noticed	slightly	moderately	strongly	very strongly
☐ x 0 = 0	☐ x 1 = ☐	☐ x 2 = ☐	☐ x 3 = ☐	☐ x 4 = ☐	☐ x 5 = ☐

Total score ☐ **out of 35**

Development/Reference: R. Freynhagen, R. Baron, U. Gockel, T.R. Tölle / Curr Med Res Opin, Vol.22, No. 10 (2006)
©2005 Pfizer Pharma GmbH
painDETECT questionnaire, ©2005 Pfizer Pharma GmbH, used with permission.

painDETECT

SCORING OF PAIN QUESTIONNAIRE

Date: ____________ **Patient:** Last name: _______________ First name: _______________

Please transfer the total score from the pain questionnaire:

Total score [|]

Please add up the following numbers, depending on the marked pain behavior pattern and the pain radiation. Then total up the final score:

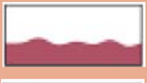	Persistent pain with slight fluctuations	0	
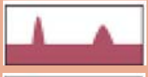	Persistent pain with pain attacks	- 1	if marked, or
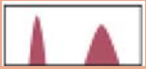	Pain attacks without pain between them	+ 1	if marked, or
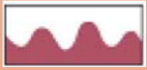	Pain attacks with pain between them	+ 1	if marked
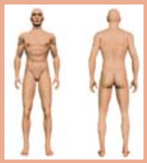	Radiating pains?	+ 2	if yes
	**Final score**	[|]	

Screening Result

Final score

nociceptive	unclear	neuropathic
0 1 2 3 4 5 6 7 8 9 10 11 12	13 14 15 16 17 18	19 20 21 22 23 24 25 26 27 28 29 30 31 32 33 34 35 36 37 38
A neuropathic pain component is unlikely (< 15%)	Result is ambiguous, however a neuropathic pain component can be present	A neuropathic pain component is likely (> 90%)

This sheet does not replace medical diagnostics.
It is used for screening the presence of a neuropathic pain component.

Development/Reference: R. Freynhagen, R. Baron, U. Gockel, T.R. Tölle / Curr Med Res Opin, Vol.22, No. 10 (2006)

©2005 Pfizer Pharma GmbH
